# Research-based narrative videos to reduce stigma: insights from older women living with HIV

**DOI:** 10.3389/fpubh.2025.1620599

**Published:** 2025-08-13

**Authors:** Sadie B. Sommer, Julie V. Barroso

**Affiliations:** Vanderbilt School of Nursing, Center for Research Development and Scholarship, Nashville, TN, United States

**Keywords:** HIV-related stigma, older WLWH, stigma reduction, narrative video intervention, focus group, internalized stigma, Southern United States

## Abstract

**Introduction:**

Older women living with HIV (WLWH) experience a heightened burden of stigma, compounded by age, gender, social isolation, and depression. Despite growing recognition of these challenges, few stigma reduction interventions are specifically designed for this population, particularly in the Southern United States.

**Methods:**

We explored the acceptability, personal relevance, and perceived effectiveness of a stigma reduction video series tailored for older WLWH. The videos were developed based on findings from a prior qualitative metasynthesis and reflected the lifecycle of stigma across key themes. A single in-person focus group with 18 older WLWH was conducted at an HIV service organization in the Southern U.S. Participants viewed the videos and provided feedback through structured discussions. Qualitative descriptive analysis and thematic analysis were used to identify key themes.

**Results:**

Participants, predominantly African American/Black women with a mean age of 59 years and an average of 24 years since HIV diagnosis, responded positively to the videos. Four major themes emerged: (1) resurfacing memories of early HIV-related stigma; (2) the persistence of internal and perceived stigma; (3) growth, acceptance, and advocacy with aging; and (4) preferences for video format and content, including a desire for more dynamic visuals, expanded educational material, and representations of the full journey of living with HIV from diagnosis to long-term survivorship.

**Discussion:**

Brief, narrative-driven videos rooted in lived experiences show promise as a stigma reduction strategy for WLWH. Future interventions should incorporate multigenerational perspectives, expand educational content, and leverage visually engaging formats to enhance relevance and impact.

## 1 Introduction

In the United States, more than half (54%) of people diagnosed with HIV are aged 50 and older (1), and women aged 35 and older account for 58% of new diagnoses among women (2). However, there are currently no national statistics reporting the number of older women living with HIV (WLWH), highlighting a critical gap in research and surveillance efforts. What is known is that older WLWH experience a higher burden of comorbid diseases (3) and are particularly vulnerable to social isolation and depression (4–6). Additionally, they face unique challenges, including HIV-related stigma, often compounded by ageism and sexism, with women being more adversely impacted than men (7).

Older WLWH occupy a distinct and often overlooked space, where the effects of aging intersect with the long-term realities of living with HIV. Physiologically, aging with HIV can accelerate the onset of comorbidities such as cardiovascular disease, osteoporosis, and neurocognitive disorders (8). Additionally, studies have shown that as older WLWH transition through menopause, they experience elevated rates of somatic and psychological symptoms that can further compromise physical and emotional wellbeing (9). Psychosocially, older WLWH frequently encounter compounded stigma related to both HIV and age, which can contribute to social isolation, depression, and diminished quality of life (10, 11). These experiences are often exacerbated by intersecting forms of discrimination, including ageism, sexism, and racism, which may heighten feelings of invisibility, shame, or reduced self-worth (12–14). Additionally, systemic barriers, such as socioeconomic disadvantage and limited access to age-appropriate, HIV-competent healthcare, further compound the challenges faced by this population (15).

WLWH are particularly vulnerable to stigma due to prevailing gender stereotypes that blame women for their HIV status (16) and societal expectations related to motherhood, sexuality, and morality (17). Regionally, WLWH in the Southern United States face compounded experiences of stigma and social isolation due to culturally conservative views regarding HIV transmission and gender roles (18). Among older WLWH, this stigma is often intensified, as expectations around caregiving, motherhood, and moral responsibility become more deeply ingrained with age, amplifying the perceived transgression (19). This intensified stigma often persists into older adulthood, adversely affecting quality of life and engagement in care (20, 21).

In 2023, we published *A Qualitative Metasynthesis of Stigma in Women Living with HIV in the United States* (22) to examine the current state of stigma since the previous metasynthesis (23) and to guide the development of a stigma reduction intervention specifically tailored to the experiences of WLWH. The gender inequities observed in our metasynthesis mirror findings reported by others, highlighting the ongoing pervasiveness of both felt and enacted stigma, as well as the gender-linked intensification of HIV stigma (19, 24). Such enduring inequities underline the need to better understand and address the experiences of stigma among older WLWH. For older WLWH, mitigating stigma is particularly critical to improving psychosocial wellbeing and reducing health inequities in this growing demographic (25).

In our previous work, we conducted a randomized clinical trial (RCT) to test the feasibility, acceptability, and preliminary efficacy of a video-based self-management intervention, *Maybe Someday: Voices of HIV-Positive Women* (26). The results demonstrated significant improvements in self-esteem, reduced internalized stigma, and enhanced coping self-efficacy. However, over the past decade, the media landscape has shifted dramatically toward digital platforms, characterized by on-demand access to content across various devices and gravitation toward shorter video formats (27, 28). Therefore, this video intervention was adapted to reflect changes in both media consumption and stigma for WLWH.

Despite growing awareness, stigma interventions specifically targeting WLWH remain limited (29). Some exceptions include the UNITY intervention, a stigma-reduction program for African American WLWH (30), and early applications of technology, such as our use of an iPod Touch device for stigma reduction among WLWH (31). Psaros et al. also piloted an app to promote resilience among older WLWH (32). A recent review highlights the critical need for social support and stigma reduction across the lifespan for older adults living with HIV (33). However, interventions specifically designed for older WLWH remain scarce (34, 35). Building on previous findings and addressing critical gaps in the literature, this study explored the acceptability, personal relevance, and perceived effectiveness of a series of stigma-reduction videos among older women living with HIV (WLWH) in the Southern U.S., with the aim of reducing stigma across the lifespan.

## 2 Methods

### 2.1 Design

**Figure 1:**
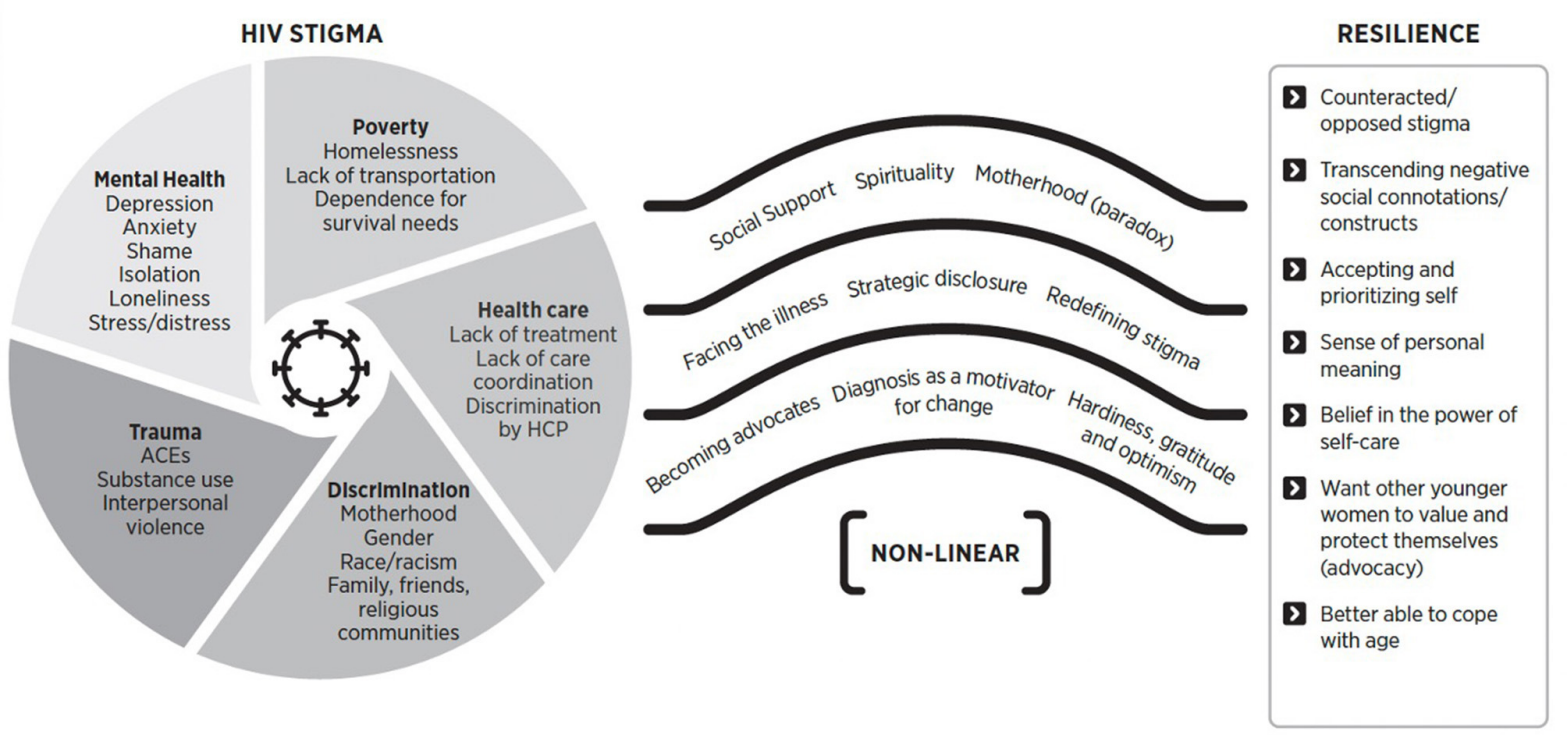
Resilience in transcending stigma (22). HPC, healthcare provider; ACEs, adverse childhood experiences. Reprinted with permission from (22), licensed under CC BY.

The intervention videos were developed directly from the findings of a qualitative metasynthesis, “*A Qualitative Metasynthesis of Stigma in Women Living with HIV in the United States”* (2023), which synthesized data from 43 qualitative studies involving 1,118 women across the U.S. The goal of the metasynthesis was to examine the current state of HIV-related stigma experienced by WLWH and to inform the development of targeted stigma reduction interventions. Through the analysis, three major thematic categories were identified: (1) Intersecting Sources of HIV-Related Stigma, (2) Non-linear Pathways to Transcending Stigma, and (3) Resilience. These themes and their corresponding subthemes served as the conceptual framework for the video scripts. A visual representation of the findings from this metasynthesis is provided below (Figure 1).

Narrative arcs identified in the metasynthesis were interpreted into brief video scripts that reflect the lived experiences, coping strategies, and stigma navigation pathways of WLWH. Three videos—each under 5 min—were scripted and portrayed by professional actors. Several considerations guided the decision to use actors. First, the scripts were intentionally constructed from the collective experiences of WLWH in the U.S. and were therefore inherently not representative of singular experience. Recent research on stigma reduction in mental health contexts has shown that brief video interventions featuring professional actors, when informed by individuals with lived experience, are equally effective in reducing stigma as those featuring the individuals themselves (36). Moreover, we believe the complexity of personal narratives deserves more time than short-format videos allow, risking the loss of coherence and meaning when edited. In contrast, scripted performances can be refined in advance to ensure clarity, emotional impact, and fidelity to core themes. Lastly, using actors offers a respectful and effective way to convey authentic experiences while allowing individuals to maintain privacy and anonymity when preferred. Summaries of the scripts are presented below.

Script 1: Stigma is a lived reality but also a space for connection, mentorship, and growth.

Video 1 features a conversation between two women living with HIV following a support group meeting. Speaker 1, who has lived with HIV for over 20 years, provides guidance, empathy, and encouragement to Speaker 2, a younger woman diagnosed 4 years ago who at times struggles to manage her care and cope with stigma. Their dialogue touches on personal experiences with stigma, from family rejection to healthcare discrimination, and explores their perceptions and reactions to those experiences. Speaker 1 shares her journey toward self-acceptance and resilience, gently helping Speaker 2 process her own feelings and begin to claim her sense of identity and agency. The conversation ends on a hopeful note, with Speaker 1 affirming Speaker 2′s growing sense of self and encouraging her to find her voice.

Script 2: Stigma is a personal journey marked by struggle, spiritual grounding, and transformation.

Video 2 is a first-person monolog capturing the inward and emotional process of a woman living with HIV. She recounts the pain of judgment and betrayal, even from those meant to support her, and the loneliness and fear she faced following her diagnosis. Through this hardship, she leans on spirituality and self-acceptance. Over time, she finds genuine love and support but understands that healing had to begin with loving herself. She accepts her HIV status as part of her life journey and now speaks from a place of hope and purpose, determined to be present for her family and to inspire others who may be struggling as she once did. The monolog is accompanied by curated stock imagery from Getty Images, a professional stock photography provider, selected to visually align with the spoken content.

Script 3: HIV is a manageable, chronic condition, and stigma is a social construct, not a personal experience.

Video 3 is a first-person monolog that features a younger woman reflecting on her HIV diagnosis. Her stance is empowered and fact-based, and she frames her diagnosis not as a moral failure but as a consequence of the common experience of condomless sex. She speaks about the persistence of stigma despite increased awareness and medical advancements and reflects on how her own pre-diagnosis assumptions about HIV were rooted in misinformation. Now, she is focused on educating others, promoting the importance of ART adherence, and advocating for dismantling the cultural stigmatization of HIV. As with the second video, the monolog is accompanied by moving stock imagery that visually aligns with the spoken content.

Table 1 presents the themes from the metasynthesis as they are presented across the three stigma-reduction video scripts.

**Table 1:** Placement of metasynthesis themes in stigma-reduction video scripts.

**Theme**	**Subtheme**	**Script 1**	**Script 2**	**Script 3**
Intersecting sources of HIV-related stigma	Mental Health	Speaker 2 expresses stress, shame, and emotional distress tied to her diagnosis	Speaker describes deep fear, loneliness, and emotional pain post-diagnosis	Minimal emotional distress; reflects more on past misconceptions than internal shame
Intersecting sources of HIV-related stigma	Trauma	Speaker 1 alludes to past trauma and emotionally painful experiences, though not explicitly	Mentions betrayal and emotional trauma; hints at past adversity without detail	
Intersecting sources of HIV-related stigma	Discrimination	Speaker 2 experiences family rejection; Speaker 1 shares stigma from medical professionals	Judgment from family and healthcare professionals is referenced	Acknowledges societal stigma but does not describe direct personal discrimination
Intersecting sources of HIV-related stigma	Poverty	Speaker 2 mentions difficulty attending support groups; transportation barriers implied	Discusses nondescript past hardships	
Non-linear pathways to transcending stigma	Social support	Strong peer support between the two speakers; mentorship dynamic is central	Mentions finding “truest friends” and being surrounded by love	Support will come from her educated community
Non-linear pathways to transcending stigma	Spirituality	Not spiritually focused	Speaks openly about relying on God during her darkest times	Not spiritually focused
Non-linear pathways to transcending stigma	Motherhood	Speaker 2 is motivated by her daughter to stay engaged in care	Wants to live for her kids and grandchildren	N/A
Non-linear pathways to transcending stigma	Facing the illness	Speaker 1 shares how she used to avoid care; Speaker 2 discusses not wanting to talk about HIV	Reflects on being scared and how it took time to feel okay	Speaks matter-of-factly about having HIV and moving forward
Non-linear pathways to transcending stigma	Strategic disclosure	Speaker 1 encourages selective disclosure; validates not telling everyone		Open with her status
Non-linear pathways to transcending stigma	Redefining stigma	Speaker 1 reframes others' reactions as ignorance; encourages empowerment	Accepts HIV as part of her life and integrates it into her story	Frames stigma as outdated and rooted in ignorance
Non-linear pathways to transcending stigma	Becoming advocates	Speaker 1 hints Speaker 2 may eventually become an advocate	Wants to give hope and help others who are struggling	Advocates for awareness and normalization; believes in public education
Non-linear pathways to transcending stigma	Diagnosis as a motivator for change		Diagnosis became a turning point in her personal growth	Uses diagnosis as a platform to educate and advocate
Non-linear pathways to transcending stigma	Hardiness, gratitude and optimism	Speaker 1 demonstrates hardiness by sharing her story and optimism, telling Speaker 2 she's ‘already doing it'	Shows optimism and a future-focused mindset	Confident, calm, and future-focused; does not express emotional turmoil
Resilience	Counteracted/opposed stigma	Speaker 1 speaks out against stigma and educates others	Speaks against unjust treatment; reframes HIV as something she can live with	Describes thick skin; does not internalize judgment
Resilience	Transcending negative social connotations/ constructs	Speaker 1 challenges negative stereotypes directly with reframing	Rejects shame, frames HIV as a part of her identity, not a moral failure	Challenges assumptions; normalizes HIV through facts and confidence
Resilience	Accepting and prioritizing self	Speaker 1 models self-acceptance and encourages Speaker 2 to embrace all parts of herself	States self-love was essential to accepting others' love	States she is fine the way she is, with or without a cure
Resilience	Sense of personal meaning	Speaker 1 expresses meaning from her long journey and her role as a mentor	Sees her experience as meaningful and wants to use it to help others	Believes education is key to survival and empowerment
Resilience	Belief in the power of self-care	Not explicitly stated but implied through maintaining care and resilience	Believes in the power of health and inner peace	Believes in empowerment through education, especially for young people
Resilience	Want other younger women to value and protect themselves (advocacy)	Speaker 1 offers indirect encouragement to share her story	Speaks directly to younger women in need of hope and support	Wants young people to understand risk and reality of HIV
Resilience	Better able to cope with age	Speaker 1′s journey of 24 years shows ability to cope with age and time	Age and experience help her feel stronger and more grounded	Not applicable—speaker is younger and more present-focused

### 2.2 Recruitment and inclusion criteria

The Vanderbilt University Medical Center Institutional Review Board approved all phases of this study. Participants were enrolled if they met the following inclusion criteria: (1) self-identified as a woman (cis or transgender); (2) living with HIV or AIDS; (3) being aged 18 or older; (4) were able to speak and read English; and (5) were able to provide informed consent. We used convenience and snowball sampling to recruit eligible women. Recruitment occurred at an AIDS service organization located in the Southern U.S. After meeting with staff to discuss the study objectives, inclusion criteria, and compensation, and to field any questions, staff selected a date based on their availability and informed their clients who were eligible about the focus group. We also supplied fliers to be placed around the center to assist with recruitment. If individuals were interested, the staff provided more information about the date, time, and location of the focus group. The staff re-contacted all participants 1 week prior to the focus group to ensure prospective participants were still able to attend or had any questions about the group.

### 2.3 Data collection

We conducted a single, in-person focus group with 18 WLWH on January 15, 2025, lasting ~1 h. A qualitative methodologist with extensive stigma-related expertise facilitated the focus group. Additional study personnel trained in qualitative methodology were present to assist with detailed note-taking and logistics.

The discussion guide was developed by the research team to support open-ended feedback on each of the three videos. Questions focused on personal relevance, relatability, and suggestions for improvement, with additional questions addressing broader impressions of the format, visuals, and delivery preferences. While no formal guidelines are specifically tailored to focus groups evaluating video content, established best practices were applied to this context (37, 38). Additionally, the structure of the guide was informed by the study's aims, expertise in qualitative methodology and working with stigmatized populations.

Prior to the start of the focus group, participants completed a written consent form and a brief sociodemographic questionnaire. After each participant had given their consent, study personnel introduced themselves, and the facilitator outlined the purpose of the discussion, explained the confidentiality of the data collected, and reviewed the compensation for participation. Participants were compensated with a $50 gift card. The focus group was conducted in a familiar, community-based setting where participants had access to trusted staff with whom they had established relationships. A structured discussion guide was then used to prompt conversation and ensure consistent feedback across all three videos. Before each video, we provided a brief, plain-language description of its content and setting, then participants were shown the video. After each video, they were asked to share which elements of the videos they related to, which parts they did not, and how the content could be improved to more accurately reflect their experiences and be most impactful to other WLWH. After viewing the series, they were also prompted to discuss their reactions to the use of actors, the length, the images and format, what they liked and disliked and why, and their preferences for setting delivery platforms.

### 2.4 Data management and analysis

We summarized participants' sociodemographic characteristics using descriptive statistics. Study personnel manually entered data from the paper surveys completed during the in-person focus group into REDCap (Research Electronic Data Capture), a secure, web-based software platform designed to support data capture for research studies (39). The paper copies of the forms are stored in a locked drawer in a secure location. The focus group session was audio-recorded, transcribed verbatim, reviewed for accuracy, and stored securely on an encrypted drive.

We employed a qualitative descriptive design (40) as the methodological framework, allowing us to remain data near (41) and avoid abstract theorizing. Using thematic analysis (42), we analyzed the focus group transcript to identify key themes in participants' responses. This approach enabled us to capture recurrent concepts while staying grounded in participants' own language and perspectives. Two researchers (JB, SS) independently read the transcripts. One author (SS) conducted a detailed, line-by-line review of the transcript to identify and extract text segments relevant to both the original study aims (e.g., stigma experiences, reactions to the videos) and unanticipated but salient themes that emerged during the discussion (e.g., enduring impact of early stigma, internalized stigma, reflections on aging with HIV). These segments were compiled into a structured data table with contextual notes to preserve meaning. The second author (JB) reviewed the full transcript alongside the extracted data to confirm completeness and contextual fidelity.

The two authors then jointly reviewed and organized the verified excerpts through an iterative process of theme development. Thematic categories were refined through discussion and triangulated with field notes, video content, and the conceptual framework from the prior metasynthesis. This process ensured that identified themes accurately reflected participant perspectives. To enhance authenticity and depth, representative direct quotations were included throughout the results. To enhance contextual richness and preserve the authenticity of participants' voices, we incorporated direct, uninterpreted quotations throughout the findings. All the data have been retained for confirmability.

## 3 Results

### 3.1 Demographic characteristics

The study included 18 female participants with a mean age of 59 years. The majority were African American/Black (83.3%) and heterosexual (94.4%). On average, participants had been living with HIV for 24 years. Most participants (72.2%) lived below the federal poverty level (FPL) and had attained at least a high school diploma or GED. In addition to providing a range of standardized options, participants had the opportunity to write in a response if their demographic characteristic was not listed. The table includes only the options selected for reporting and does not represent an exhaustive list of all possible responses. Demographic characteristics for the study participants can be found in Table 2.

**Table 2:** Demographic characteristics of participants (*N* = 18).

**Variable**	***N* (%)**
**Gender identity**	
Female	18 (100)
Mean age	59
Age Range	40–75
**Race**	
African American/Black	15 (83.3)
White non-Hispanic	2 (11.1)
Mixed race	1 (5.6)
**Sexual orientation**	
Heterosexual/straight	17 (94.4)
Bisexual	1 (5.6)
Mean years since diagnosis	24
Range of years since diagnosis	2–36
**Highest level of education**	
Some high school	5 (27.8)
High school degree or GED	6 (33.3)
Some college	4 (22.2)
Associate (2-year) degree	2 (11.1)
Bachelor's (4-year) graduate	1 (5.6)
**Employment status**	
Full-time	6 (33.3)
Part-time	1 (5.6)
Unemployed/looking for work	1 (5.6)
Disabled	7 (38.9)
Retired	3 (16.7)
Household mean	2
Household range	1–4
Above FPL	5 (27.8)
Below FPL	13 (72.2)

GED, Graduate Equivalency Degree; FPL, Federal Poverty Level; “Household mean” refers to the average number of individuals per household.

### 3.2 Memories of HIV-related stigma

The focus group evoked strong personal memories, resurfacing stigmatizing past experiences.

#### 3.2.1 Stigmatizing experiences

After viewing the first video, a woman stated, “*It brought some memories when I first was diagnosed, and that I haven't thought of in over 35 years, and I've come a long way from the'90s till now.”* They shared stigmatizing experiences in healthcare settings, including being required to register with the health department after diagnosis and facing prejudiced treatment from medical staff. A participant describes, “*She started putting on double gloves, and my mama politely said, ‘Honey, move. You don't have to do all of that. You can't catch it.' My mom kind of started angrily educating her, but for me, the damage had been done*.”

#### 3.2.2 Fear and misinformation

The videos also resurfaced the fear and misinformation surrounding HIV in earlier decades. One participant became emotional as she remembered believing she could spread HIV through shared objects like toilet seats and tableware when she was first diagnosed. She described her own prejudice before her diagnosis. “*That was what I would've done myself. If you would've come out positive to me, I would've been afraid to go near you back at that time.”*

### 3.3 Internal and perceived stigma

The videos prompted discussion on the impact of HIV-related stigma, both from within themselves and from external sources.

#### 3.3.1 Internal stigma

Participants noted that internal stigma can be as powerful as external stigma. One woman described feeling out of place in a high-end medical office—not due to mistreatment but because of her own internalized discomfort. She describes, “*You walked in, everything was white and bright. It was this beautiful, serene kind of place, and in my mind…I started feeling some kind of way, not because of how they treated me. It is just the way I felt—a little uncomfortable.”*

#### 3.3.2 Perceived stigma

Many participants reflected on the weight of others' opinions. A participant asked, “*Still now. I'm 57 years. Why would I care about my neighbor or whomever who's not in my house, not paying no bills? I don't even know you. Why would I even worry? But we do, we do.”* A woman responded, “*I think it's just human nature. We care about what people think about us and what they say. Even though we say, ‘I don't care, I don't care.' Words hurt.”* The women's reactions to stigmatizing experiences ranged from indifference to confrontation, but many acknowledged the difficulty of truly letting go of others' judgment. A participant stated, “*I want to be to that place where you don't care…I don't know if we'll ever get out of that.”*

### 3.4 Aging with HIV: advocacy, disclosure, and acceptance

The focus group discussions highlighted the journey of aging with HIV as one of learning, self-acceptance, and advocacy while navigating ongoing stigma.

#### 3.4.1 The power of education

Participants stressed the importance of education for themselves, others, and the next generation. One woman shared, “*I think education is the key to really living a long life with it. Once you know how it's in your body and know how it affects your immune system, the better.”* Education was also seen as a duty. One woman stated, “*It's my responsibility to educate people because ignorance is real, and if they don't know, they don't know. So, every opportunity I get, I'm a big mouth. I help educate people.”* Others viewed the videos as tools for prevention. One participant explained, “*If I can stop'em from having to go down one or two of those hard roads, then my being diagnosed and having to go through that stuff are not in vain.”* Another stressed persistence: “*As soon as you think that you need to stop talking, you need to talk more… you might not get ‘em all, but you'll get somebody that it might hit home.”*

#### 3.4.2 Balancing disclosure and trust

The videos prompted discussion around disclosure. Some women were open about their status, with one saying, “*I'll have a conversation with somebody on the bus. It don't matter because I'm not HIV. It's just something I have.”* Another added, “*I don't care who knows it because I'm living longer. I've overcome the bad part of it.”* However, others felt unsafe disclosing outside trusted spaces. One woman admitted, “*I don't talk freely at all, but I'm very confident with who I am in this group, and I'm confident in the real world, too. But I still don't trust people. That stigma is a killer.”* Another participant shared that she will defend a “friend” with HIV as a way to conceal her own status, adding, “*I feel like a liar in the real world, and I am.”* Others reassured her, emphasizing disclosure is their choice: “*This is your baby. You don't have to share your baby with anyone you don't want to. And they have to be deserving.”*

#### 3.4.3 Finding acceptance

In response to a video sharing positive messages of growth, participants reflected on their paths. One woman shared, “*It took me around 16 years to accept that I am actually HIV positive before I was able to move on from some hurt and emotional pain. So, you have to accept it first.”* Another stated, “*I got tired of living behind the mask. Now I don't have to do that anymore, and I refuse to.”* Diagnosed only a year and a half ago, one participant acknowledged that she had not yet reached the same level of acceptance as others in the group. The group responded with support and encouragement. One reassured her, “*It takes time. I wasn't talking as much as you are now.”* Another added, “*I would have never guessed…the way she talks in groups, she's so strong and confident with the disease.”* Participants reiterated that self-acceptance is a process shaped by time and experience. One woman reflected on this journey, stating, “*But that takes time, longevity. And I believe spirituality has a lot to do with it as well.”*

### 3.5 Direct feedback

Direct feedback on the videos, highlighting aspects the women appreciated and areas for improvement.

#### 3.5.1 Content suggestions

The second video's message of self-love resonated with some participants and spurred discussion on the distinction between self-love and self-acceptance. A participant suggested using the word “acceptance” instead of “love”, noting, “*If you don't accept what have happened to you, no matter how much you love yourself, you'll never move on.”* Some suggested creating a series of videos showing different stages of the HIV journey—from diagnosis to long-term survival—to help illustrate personal growth over time. The group also suggested more comprehensive educational content in the videos. They suggested covering topics such as HIV basics, condom use, and U=U (Undetectable = Untransmittable). Some also felt that HIV should be discussed in the context of other STDs rather than as a separate issue.

#### 3.5.2 Visual preferences, length, and format

Participants preferred the light, optimistic imagery in the second and third videos and found them more engaging than the still frames in the first. They felt the first video lacked visual appeal, noting that the setting was plain and the actors did not seem connected. A participant noted, “*The information was relevant and interesting. It was just something about, maybe it wasn't, maybe not enough pictures, or it was just the setting.”* Participants liked the length of the videos but again preferred the second and the third to the first, noting, “*Especially the first one on my phone was kind of long, and it was kind of long, not boring.”* When asked about other potential formats, such as animation or graphic novels, participants suggested those formats may appeal to younger audiences but expressed concern that a comic book might seem too lighthearted for the subject matter. When asked whether they would watch the videos alone or in a support group, participants preferred group viewing.

## 4 Discussion

Using a focus group of older women living with HIV (WLWH) in the Southern U.S., this article explored the acceptability, personal relevance, and perceived effectiveness of a stigma-reduction video series focused on the lifecycle of stigma. Grounded in a prior metasynthesis of qualitative literature on HIV-related stigma, the videos were designed to reflect the lived experiences of WLWH, challenge stigma, and foster discussion and resilience. Focus group findings offer insight into the ongoing burden of HIV stigma, the evolving process of aging with HIV, and the potential of visual storytelling as a meaningful tool for stigma reduction, suggesting early promise for future application.

The videos resurfaced powerful memories of early HIV-related stigma among participants, many of whom were diagnosed decades ago. Although long past, these moments remained emotionally vivid, highlighting the profound and lasting impact of institutional stigma in healthcare settings, also documented in contemporary research (53). These reflections revealed how early experiences of stigma continue to shape the identity and the emotional evolution of aging with HIV (43). This response underscores the enduring toll of HIV stigma and supports contemporary research showing that internalized stigma can persist for older WLWH even as HIV becomes increasingly normalized within clinical and social contexts (20). Feelings of not belonging or discomfort, even in the absence of overt discrimination, reflected how early experiences with stigma continue to influence self-perception. Despite years of actively advocating and opposing stigma, many participants acknowledged the difficulty of fully shedding concern for societal judgment. This tension may be heightened for older WLWH, who continue to carry the weight of historical and intersectional stigma (14, 16).

Age emerged as a powerful lens through which participants engaged and interpreted the videos. With an average of 24 years since diagnosis, many women described a long arc of adjustment from initial trauma to self-acceptance and advocacy, mirroring findings from research and our metasynthesis, which suggests older WLWH often demonstrate emotional adaptation and resilience over time, despite the compounded stigma of aging and HIV (44, 45). The videos served as a springboard for this dialogue, helping participants articulate how aging with HIV is not only about surviving but about growing into a fuller sense of purpose and self. For many, aging with HIV has brought a deeper understanding of the condition and a strong sense of responsibility to educate others, especially younger women. Education was seen as empowering and essential, and the videos reinforced the value of sharing knowledge to prevent future stigma and hardships (46, 47).

Direct feedback on the videos revealed that visual presentation played a key role in how participants received the videos. Videos 2 and 3, which paired emotionally resonant narratives with stock imagery, were viewed as more engaging and impactful than the still frames in Video 1. This preference aligns with research indicating a broader shift toward concise, visually stimulating video formats that enhance viewer engagement (48). Although such formats are often associated with younger audiences, our findings suggest that older viewers also appreciate and respond well to shorter, visually rich content. Participants also offered suggestions for enhancing the videos' impact. They recommended incorporating more educational content, particularly around basic HIV facts, to expand their usefulness for both WLWH and broader public audiences. Several emphasized the value of structuring future videos around different phases of the HIV journey, from diagnosis through long-term survivorship, to better reflect the full range of experiences and the evolving nature of living with HIV.

A major strength of this work is the integration of the metasynthesis with intervention development to root the intervention in a collective experience. By translating synthesized themes into scripted narratives, the videos conveyed broad yet nuanced concepts and perspectives. Additionally, the focus group allowed content to be developed with the target audience rather than for them, supporting the acceptability and relevance of the content. We previously developed and tested a longer-form video intervention to address HIV-related stigma among WLWH, which demonstrated reduced internalized stigma and improved self-efficacy in coping. Building on that success, this study explores a short-form, narrative-driven approach. While growing evidence suggests that brief, narrative-driven video interventions grounded in authenticity can effectively engage participants and reduce mental health stigma (49, 50), this study is, to our knowledge, the first to explore a short-form video intervention specifically for reducing HIV-related stigma among WLWH. Additionally, this holds tremendous potential due in part to its ability to reach beyond physical sites to engage individuals where they feel safe, a necessary component of any intervention targeting stigmatized populations (51). Furthermore, it has the capability to continue uninterrupted during disease outbreaks, like the COVID-19 pandemic, without restriction (52).

Nonetheless, several limitations should be acknowledged. The sample, while representative of diverse life experiences, was regionally bound to the Southern U.S. and may not reflect the perspectives of WLWH from other cultural or geographic backgrounds. To this demographic point, we did not capture the impressions of younger WLWH, and the interventions would benefit from incorporating their perspectives to produce the suggested educational content in the phases of diagnosis. Additionally, the single-session format may have influenced group dynamics, potentially amplifying agreement or limiting dissenting views that may have emerged in subsequent sessions. Finally, although no participants reported distress during or after the focus group, the potential for emotional discomfort should be acknowledged. The videos prompted some participants to recall painful or stigmatizing experiences from earlier in life. However, encouraging this type of reflection was a central aim, as acknowledging and validating these experiences is essential to reducing stigma and fostering healing. While the setting and procedures were designed to support emotional safety, future implementations should continue to consider the risk of resurfacing difficult memories and ensure that appropriate support is available when the videos are used in group or individual settings.

## 5 Conclusion

Our study highlights the potential of brief, narrative-based videos to engage WLWH in meaningful reflection and dialogue about HIV-related stigma. The participants' responses revealed not only the emotional resonance of the content but also the enduring impact of stigma across the lifespan. Participants emphasized the need for future interventions to reflect the full arc of the HIV journey, from diagnosis to long-term survivorship, and to include more educational content. They also offered clear preferences for engaging, visually dynamic formats. As the HIV population continues to age, there is a growing need for interventions that honor the complexities of long-term survivorship, address persistent stigma, and incorporate the voices of older WLWH (13, 32). Future work is needed to build on these findings by incorporating multigenerational perspectives, expanding educational content, and refining the visual presentation of video materials based on participant feedback. After refinement, we aim to expand this intervention to include younger WLWH and participants from other regions in the U.S. The video format is brief, cost-effective, and optimized for virtual delivery across multiple platforms, making it highly scalable and adaptable to diverse settings. The videos are well-suited to complement existing peer support programs, care engagement apps, and support groups by fostering reflection, discussion, and connection. As such, they could be readily integrated into care management across HIV services, community-based organizations, and digital platforms. Following refinement of the videos based on this formative work, longitudinal research will be necessary to assess their sustained impact.

## Data Availability

The raw data supporting the conclusions of this article will be made available by the authors, without undue reservation.
